# The Efficiency of Photodynamic Therapy in the Bacterial Decontamination of Periodontal Pockets and Its Impact on the Patient

**DOI:** 10.3390/diagnostics12123026

**Published:** 2022-12-02

**Authors:** Ioana R. Munteanu, Ruxandra E. Luca, Marius Mateas, Laura Diana Darawsha, Simina Boia, Eugen-Radu Boia, Carmen D. Todea

**Affiliations:** 1 Department of Oral Rehabilitation and Dental Emergencies, Faculty of Dentistry, “Victor Babes” University of Medicine and Pharmacy, Eftimie Murgu Square No. 2, 300041 Timisoara, Romania; 2 The Interdisciplinary Center for Dental Medical Research, Lasers and Innovative Technologies, Piaţa Eftimie Murgu Nr. 2, 300041 Timişoara, Romania; 3 Department of Mechatronics, Polytechnic University of Timisoara, Mihai Viteazu Blvd., 300222 Timisoara, Romania; 4 Laura Diamond Dent Srl, Strada Alexandru Odobescu 2, 30020 Timișoara, Romania; 5 Department of Periodontology, “Victor Babes” University of Medicine and Pharmacy, 300041 Timisoara, Romania; 6 Department of Ear, Nose and Throat, “Victor Babes” University of Medicine and Pharmacy Timisoara, 2 Eftimie Murgu Sq., 300041 Timisoara, Romania

**Keywords:** photodynamic therapy, non-surgical periodontal therapy, PCR, randomized controlled clinical study

## Abstract

Research in the field of periodontal disease continues to focus on disease-associated microorganisms, as the microbial plaque and the host immune responses are considered to be important causative factors, that are highly responsible for the progression of this disease. The purpose of this article is to compare the reduction in the number of specific periodontopathogens in two test groups according to different therapeutic approaches in periodontal disease and to show possible differences. This article is based on a prospective clinical study involving eighteen subjects with forty-four average periodontal pockets assigned to study groups treated by two different methods, SRP and SRP followed by a single PDT application. Efficiency in removing specific bacterial species was evaluated by PCR testing, at baseline and immediately after treatment. The hypothesis that using SRP + aPDT results in an increased decontamination potential was confirmed statistically, when all five specific bacterial pathogens were investigated together. When the pathogens were considered separately, two of the five microorganisms tested were significantly lower in the SRP + PDT group (*p* < 0.00), and important germ counts reductions were also observed for the other three. There is also a statistically significant relation between the pain at 48 h postoperatively and the type of treatment the patients received, as resulted from the Questionnaire Form. Our results demonstrate that aPDT, as an adjunctive treatment to conservative mechanical cleaning of root surfaces at sites affected by periodontitis, represents an effective tool in terms of reducing specific periodontopathogen germs.

## 1. Introduction

Periodontitis is an infection with multiple bacteria involvement that affects the tooth-supporting tissues destroying connective tissue and alveolar bone, and may eventually lead to loss of teeth [1]. The microbial plaque and the host immune responses are considered to be important causative factors that are highly responsible for the progression of this disease [2,3].

The research on periodontal disease continues to focus on these microorganisms. However, more recent studies have determined that the oral cavity contains much more prevalent species, referred to as the oral microbiota [4,5]. It has been shown that periodontitis patients carry a higher number of disease-associated bacteria than healthy ones. As technology advances, more sensitive techniques based on DNA amplification such as the Polymerase Chain Reaction, have been developed to assess the presence/number of microorganisms in the pockets.

During the development of periodontitis, the resident oral microbiota converts from the dominance of facultative gram-positive bacteria to an anaerobic gram-negative majority. Certain species and their combinations, including *Aggregatibacter Actinomycetemcomitans*, *Porphyromonas Gingivalis*, *Prevotella Intermedia*, *Treponema Denticola,* and *Tannerella Forsythia*, have been strongly implicated in the pathology of periodontitis [6,7,8]. Microbiota and the host inflammatory response can easily explain the local tissue destruction in periodontitis [9]. For many patients, nonsurgical periodontal therapy is often the first line of defense against further disease progression [10]. SRP is effective in removing biofilm, calculus, and endotoxins, that induce inflammation from the periodontally involved root surface, all these aiming towards restoring periodontal health. Therefore, it is considered the gold standard for nonsurgical periodontal therapy [11,12].

A modern noninvasive photochemical approach to infection control, namely photodynamic therapy, has received more attention in the treatment of oral diseases [13]. Photodynamic therapy (aPDT) is a potential strategy to eliminate infections in a specific tissue. It uses a low-power laser to activate a photosensitizer. Studies have shown the usefulness of aPDT in periodontal treatment. The aPDT offers the possibility of reducing bacterial load and eradicating periodontal pathogens [14]. 

The mechanism of photodynamic antimicrobial reactions implies that, after laser irradiation, the photosensitizer in the ground state is converted to a highly-energized triplet state to react with biomolecules. The highly reactive state of oxygen, known as singlet oxygen (^1^O2), has lethal effects on the bacterial cell, by damaging the cell membrane and the cell wall. [15,16,17].

The aim of this study was to compare the efficiency of aPDT decontamination in periodontal pockets for five important periodontal pathogens—*A. Actinomycetemcomitans*, *P. Gingivalis*, *P. Intermedia*, *T. Denticola* and *T. Forsythia*—by comparing SRP alone with a single application of aPDT in combination with SRP; and to evaluate patient feedback on this modern practice.

## 2. Materials and Methods

This study was a randomized, controlled, prospective clinical trial. Using a split-mouth design, each patient served as his/her own control.

The study was carried out at the “Victor Babes” University of Medicine and Pharmacy Timisoara, in accordance with the Helsinki Declaration of 2002. Ethical approval was obtained from the Scientific Research Ethics Committee of “Victor Babes” University of Medicine and Pharmacy Timisoara (Nr. 45/20.06.2022).

The primary outcomes were changes in the quantity of pathogens in the periodontal pocket immediately after the applied treatment and the patient’s perception of discomfort, pain, and satisfaction.

### 2.1. Participants Selection

After a verbal and written explanation of the study, participants who agreed to take part in the study signed an informed consent. Radiographs and photos of every case were registered.

Eighteen patients, aged between 28 and 46 years with a clinical diagnosis of localized chronic periodontitis, were selected, corresponding to the “Classification of periodontitis based on stages defined by severity [18] (according to the level of interdental clinical attachment loss, radiographic bone loss, and tooth loss), complexity and extent and distribution” for each stage, described as localized periodontitis with <30% of teeth involved.

Inclusion criteria:

- Men and women > 25 years
- No periodontal treatment or intake of antibiotics in the last 6 months
- At least 12 natural teeth present in the oral cavity distributed in all four quadrants
- Satisfactory individual oral hygiene (Plaque index > 25%)
- The presence of bleeding during the periodontal survey
- At least 4 teeth with at least one periodontal pocket (PD) ≥ 4 mm at initial assessment, but not more than 30% of the existent teeth
- Clinical and radiographic signs of localized chronic periodontitis [18]

Exclusion criteria:

- Pregnancy and/or lactation period
- Allergy to Tolonium chloride (Toluidine Blue Gel 0.005%)
- Any systemic conditions that could affect the progression and treatment of periodontal diseases, such as type 1 and 2 diabetes
- Alcohol abuse

### 2.2. Oral Examination

The initial visit: after filling out medical and consent forms and after a careful clinical evaluation, a total of 18 subjects were recruited for this study. Orthopantomograph assessment was one of the methods of investigation to determine the type of bone loss. All participants were photographed, supragingival scaling and professional cleaning was performed and they received instructions to perform dental hygiene at home. As home care, patients are advised to correctly brush 2 times a day and to use dental floss, without other additional products. 

The periodontal screening was performed two weeks after prophylactic therapy, according to Basic Periodontal Examination. Assessment of periodontal parameters was recorded in the periodontal chart (http://www.periodontalchart-online.com/uk/ accessed on 25 June 2022). At baseline, periodontal assessments consisted of the PI, PD, BOP, and CAL at six sites per tooth using a periodontal probe (The Colorvue™Oxford Periodontal Probe, Hu-Friedy, Chicago, IL, USA) and the enamel-cementum junction (JSC) as a reference point for PD. In addition, the radiographic examination was indicated in order to verify the loss of bone and root anatomy [19]. 

The examiner who performed all measurements was blinded to the type of treatment provided, while another examiner performed all treatment procedures.

### 2.3. Patient Perception Questionnaire 

Patients feel more secure when they have to choose between a classic and a more up-to-date treatment. With lasers becoming more and more present in modern dental offices, many people who are reluctant to accept traditional dental treatments are willingly accepting laser dentistry. People consider dentists who are using lasers as being able to perform state-of-the-art procedures that result in a more comfortable, predictable, and superior outcome [20]. The classical periodontal treatment uses mechanical scaling and root planning together with local decontamination agents and for some time the use of photobiomodulation for the prophylaxis and treatment of this disease has been available with good clinical results [21]. We developed a feedback questionnaire with 9 multiple-choice questions that are answered by the patients. The first part was conducted before the treatment session, in order to examine the patient’s knowledge of the laser technology. Another five questions were asked after the treatment was finished, in order to evaluate the postoperative treatment outcome in terms of pain, during and after treatment: 1. Have you ever followed classic periodontal treatment? 2. Have you ever followed laser assisted periodontal treatment? 3. Did you ever request laser assisted treatment? 4. Do you have confidence in the laser assisted periodontal treatment? 5. Was the classical periodontal treatment unpleasant? 6. Was the laser assisted periodontal treatment unpleasant? 7. Did you feel pain/need for antalgic medication immediately after treatment? 8. On which part did the pain appear most? 9. 48 h after treatment, on which side did the symptoms not subside? (Appendix A).

A verbal rating scale (VRS) [22] was used immediately after each treatment and another VRS after 48 h, to evaluate pain and the need for antalgic medicine. The VRS scale consisted of 5 categories: none, mild, moderate, severe, and extreme pain.

### 2.4. Sample Size Calculation

**Figure 1:**
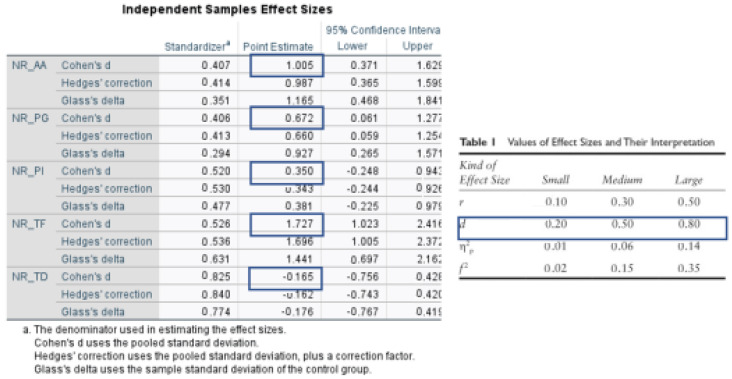
Differences: effect size based on differences between groups (e.g., Cohen’s d, Glass’ Δ, Hedges’ g, Odds ratio, and Relative risk) [23].

The Kruskal–Wallis one-way analysis (ANOVA) of variance and the Mann–Whitney U test were used to determine differences between treated areas, with different procedures. The longitudinal changes were analyzed using the Wilcoxon matched-pairs signed-ranks test. A significance level of *p*  <  0.05 was assumed for all analyses (IBM SPSS Statistics 23.0, New York, NY, USA). Based on this, 18 subjects and 44 pockets were enrolled in this split-mouth design study which would be enough to provide 80% power (Figure 1).

### 2.5. Treatment Protocol

The treatment procedures were performed in a split-mouth design: scaling and root planning (SRP) for one side and SRP + aPDT for the other side. 

The following treatment procedures were performed: scaling and root planning (SRP) for one quadrant and SRP + aPDT on the other side.

Randomly assigned, one side was selected for aPDT (study group), whilst another side served as the control group.

The chosen 2–4 periodontal pockets with PD ≥ 4 mm are mechanically instrumented under local anesthesia with Gracey curettes (Hu-Friedy, Chicago, IL, USA) and ultrasonic instruments (Piezon 250, EMS—Electro Medical Systems SA, Nyon, Switzerland). Normal saline (0.9% NaCl *w*/*v*) was used to irrigate the operative field. Non-surgical subgingival mechanical instrumentation aims at eliminating the etiologic factors on the root surface and it is considered the standard care of cause-related therapy in patients with untreated periodontitis. Subgingival mechanical instrumentation may be performed by either hand and/or power-driven instruments that result in improved clinical outcomes [24].

**Figure 2:**
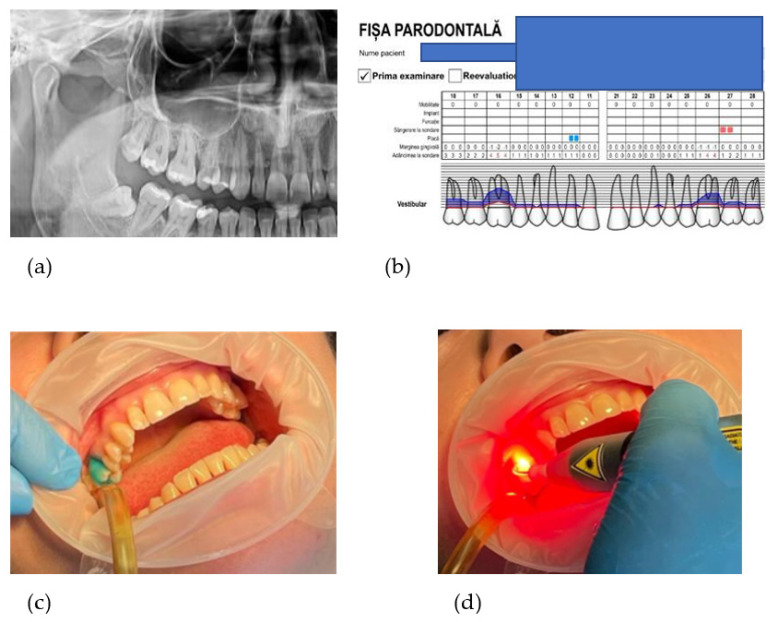
(**a**) patient X-ray showing the specific vertical bone loss in tooth 1.6; (**b**) periodontal chart showing the PD of the two selected sites for the study; (**c**) applying the photosensitizer dye liquid (Toluidine Blue Gel 0.005%; (**d**) photoactivation with the laser light at a wavelength optimized for toluidine blue solution (635 nm).

The photosensitizer dye liquid (Toluidine Blue Gel 0.005%, Cumdente) was applied with a blunt needle starting from the bottom of the periodontal pocket and gently moving coronally to avoid entrapment of air bubbles, then allowed to act for 1 min. The photosensitizer (Toluidine Blue) diffuses into biofilms, hard and soft tissue and it attaches itself to bacterial/fungal cell walls (Figure 2).

In the next stage, the pockets were rinsed with distilled water to remove excess liquid. Treatment is performed using laser light at a wavelength optimized for photoactivation of the toluidine blue solution (635 nm), with a strong 400 mW (PACT 400-Cumdente) red light laser and a PACT Light Guide Universal (white)- with spherical light emission for periodontal use, performing 3 repetitions of 10 s each, with apico-coronary oscillatory movements. 

All patients were recalled after 6 weeks in order to check their consistency with oral hygiene instructions. Clinical parameters were recorded again, 3 months after the first application of aPDT, but this result will be discussed in the future, more comprehensive study. 

### 2.6. Microbiological and Biochemical Evaluation-Crevicular Fluid Samples

Selected teeth with the greatest probing depth in two to four different quadrants were chosen, and the periodontal pockets with the deepest probing depth were selected for harvesting.

Subgingival plaque samples were collected using a commercially available kit (microIDent^®^, Hain Lifescience GmbH, Nehren, Germany) from each patient at the beginning of the session, using sterile 0.50 paper cones, for 10 s, after proper isolation [25]. All the subgingival plaque samples were collected approximately 5–7 h after tooth brushing [26].

A total of four samples were collected from each treatment pair of teeth, before and after the completion of the treatment. The samples were placed in 1.5 mL microcentrifuge tubes with 300 μL of a phosphate buffer and frozen at −20 °C until further analysis.

We established the following study groups:

○ A1 and B1 represent the negative control groups (all pockets before receiving any kind of treatment), as follows: A1—periodontal pocket before mechanical treatment (SRP), B1—periodontal pocket before mechanical treatment (SRP) and laser;
○ A2 represents the positive control group: periodontal pocket after mechanical treatment (SRP);
○ B2 represents the study group: periodontal pocket after mechanical treatment (SRP) and laser treatment (aPDT);

The commercially available kit—Micro-IDent^®^-5 is based on multiplex PCR of 16S rDNA followed by simultaneous reverse hybridization. The Micro-IDent^®^-5 has been demonstrated to be accurate for use in periodontal pathogen detection to a significant correlation with real-time PCR [27]. The micro-IDent^®^ test is based on the DNA**•**STRIP technology. The entire procedure is divided into three steps: (i) DNA extraction from subgingival plaque samples, (ii) a multiplex amplification with biotinylated primers, and (iii) a reverse hybridization.

All reagents needed for amplification, such as polymerase and primers, are included in the Amplification Mixes A and B (AM-A and AM-B) and are optimized for this test. The membrane strips are coated with specific probes complementary to the amplified nucleic acids. After chemical denaturation, the single-stranded amplicons bind to the probes (hybridization). Highly specific binding of complementary DNA strands is ensured by stringent conditions which result from the combination of buffer composition and a certain temperature. Thus the probes reliably discriminate the different sequences of the bacterial species. The streptavidin-conjugated alkaline phosphatase binds to the amplicons’ biotin via the streptavidin moiety. Finally, the alkaline phosphatase transforms an added substrate into a dye which becomes visible on the membrane strips as a colored precipitate. A template ensures the easy and fast interpretation of the banding pattern obtained.

The signals on the membrane were visually compared with a template provided by the manufacturer and scored as 0.5, 1, 2, and 3 depending on the intensity of the signal [28,29].

## 3. Results

Questionnaire assessment: All patients completed the study and healing was uneventful for all of them. There were no levels of pain or any other discomfort reported through the patient questionnaire.

The relationship between the type of treatment and the presence of pain is verified, based on the testing of categorical variables through the SQUARE CHI (CHI SQUARE) test specific to NO/YES data. It turns out that there is no relationship between treatment/location and the presence of pain during treatment. Possible cause: the patient is blinded to treatment and reports the pain based on anticipatory stress in a way that is inconsistent with reality.

However, there is a statistically significant relationship between the pain after 48 h and the type of treatment. The research hypothesis is validated, X^2^ (17, N = 13) = 5.718, *p* < 0.001. It can be observed that more patients who underwent laser treatment admit they had no pain after 48 h than in the case of classical treatment. We can therefore assess the impact of anticipatory stress, as 48 h after leaving the treatment, the level of stress no longer influences the responses and significant statistical differences appear depending on the treatment.

PCR assessment: A total of 44 samples from 18 patients were selected and assigned to either mechanical treatment (SRP alone) or SRP together with photodynamic aPDT (study group). IBM SPSS Statistics 23.0 was used for statistical analysis. One-way ANOVA test was applied using several comparison methods, Turkey, Fisher, and Hsu’s MCB. 

**Figure 3:**
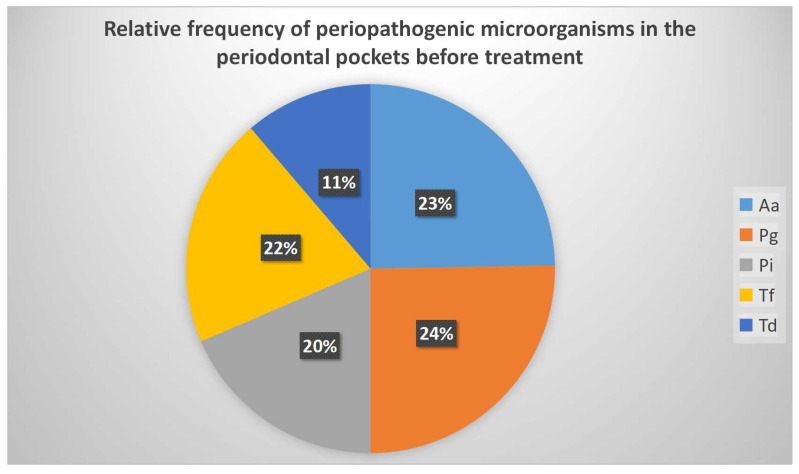
The presence of bacteria in the initial samples (A1 and B1). The colors represent different microbial complexes. (Aa) Aggregatibacter actinomycetemcomitans, (Pg) *Porphyromonas gingivalis*, (Pi) *Prevotella intermedia*, (Tf) *Tannerella forsythia*, (Td) *Treponema denticola*.

**Figure 4:**
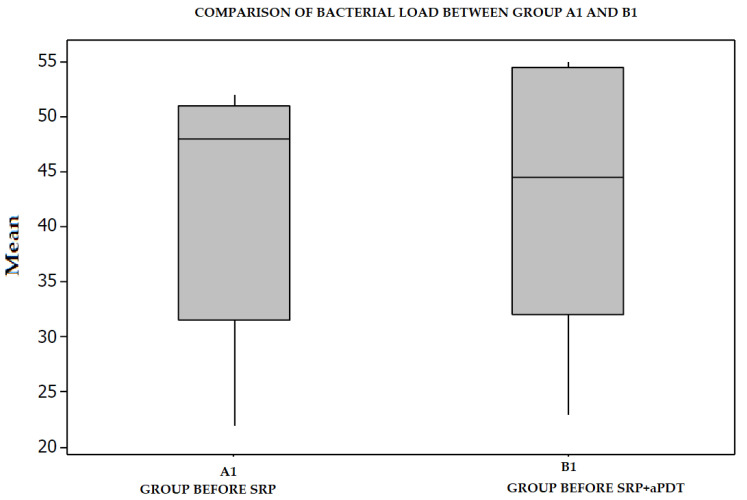
Mean bacterial count by groups, before treatment.

Following the cumulation of samples in groups A1 and B1, the frequency of periodontopathogenic microorganisms in the periodontal pockets was demonstrated as follows: *Porphyromonas gingivalis* 24%, *Aggregatibacter actinomycetemcomitans* 23%, *Prevotella intermedia* 20%, *Tannerella forsythia* 22%, *Treponema denticola* 11% (Figure 3). The difference between the two groups was not statistically significant (Figure 4).

**Figure 5:**
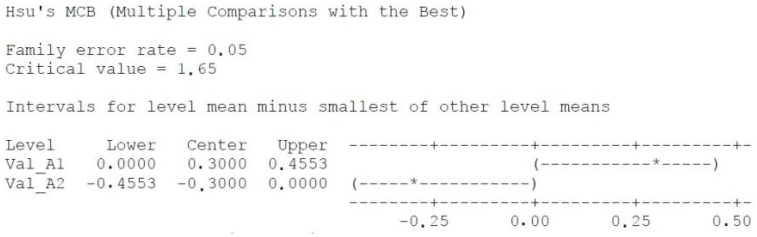
Hsu’s MCB method creates a confidence interval for the difference between each level mean and the best of the remaining level means. There is a statistically significant difference between the corresponding means. *—the OUTLIER- an unusually large or small observation. Values beyond the whiskers are outliers.

**Figure 6:**
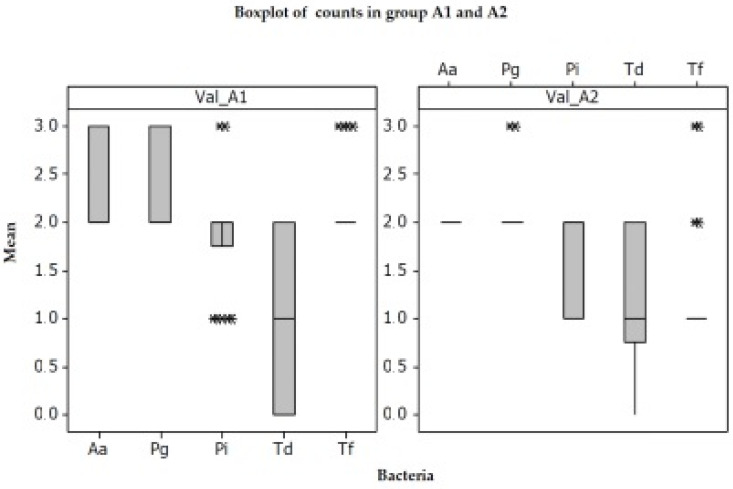
One-way ANOVA-A1 and A2 show that they are significantly different; graph shows: The best results are at Aa and Pg; Poor results are at Pi, Td, even Tf. The median is the dot in the middle of the ‘box’. The upper side of the box is the 1st quartile or 25% of the data, from the rest. The lower side of the box is the 3rd quartile, or 75% of the data, from the rest. The distance between the sides of the box is called the inter-quartile range (IQR). The distance between these is called the range.

The comparison between group A1 (before any treatment) and A2 (after SRP), reveals a significant difference between groups (*p* = 0.002), reinforcing that classical methods are effective (Figure 5), based on the detection frequency acquired from the laboratory results. Analyzing the results by every pathogen, the results show better improvement for *Aggregatibacter actinomycetemcomitans* and *Porphyromonas gingivalis* (Figure 6). 

**Figure 7:**
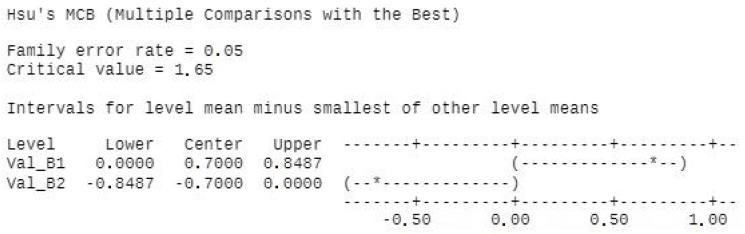
Hsu’s MCB method creates a confidence interval for the difference between each level mean and the best of the remaining level means. There is a statistically significant difference between groups B1 and B2. *—the OUTLIER- an unusually large or small observation. Values beyond the whiskers are outliers.

**Figure 8:**
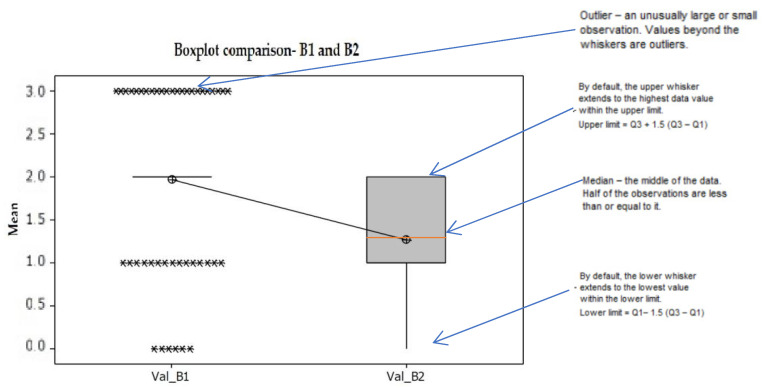
The Box plot shows the five-number summary of our set of data: including the minimum score, first (lower) quartile, median, third (upper) quartile, and maximum score. The box for B1 is actually a line (quarta Q1 and Q3 = 2 overlap). There are significant differences.The median is the red line through the middle of the ‘box’. We can see that this is just above the number 60 on the number line below. So the middle value of age is 60 years. The upper side of the box is the 1st quartile. This is the value that separates the first quarter, or 25% of the data, from the rest. The lower side of the box is the 3rd quartile. This is the value that separates the first three quarters, or 75% of the data, from the rest. The distance between the sides of the box is called the inter-quartile range (IQR). This tells us where the ‘middle half’ of the values are. The ends of the lines from the box at the left and the right are the minimum and maximum values in the data. The distance between these is called the range.

All treatment procedures reduced statistically significantly (each *p* < 0.01) the total bacterial counts. The same comparison as for conventional treatment was applied for combined treatment (SRP + aPDT) and statistics reveal significant bacterial reduction between initial group B1 and combined treatment group SRP + aPDT, B2. (Figure 7 and Figure 8). 

**Figure 9:**
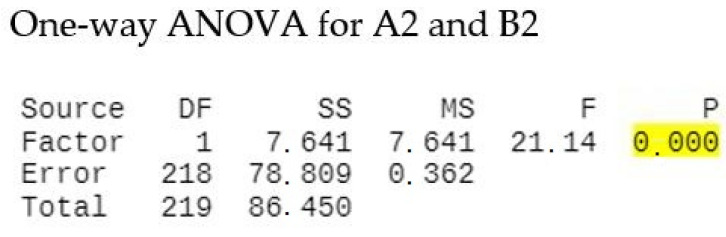
One-way ANOVA-A2 and B2 show that results are significantly different *p* < 0.05.

A one-way ANOVA test (Figure 9) compares the variance in the group means for the hypothesis that using SRP + aPDT results in an increased decontamination potential, therefore a decreased microbial count in the B2 group when compared to A2 (*p* = 0.00).

**Figure 10:**
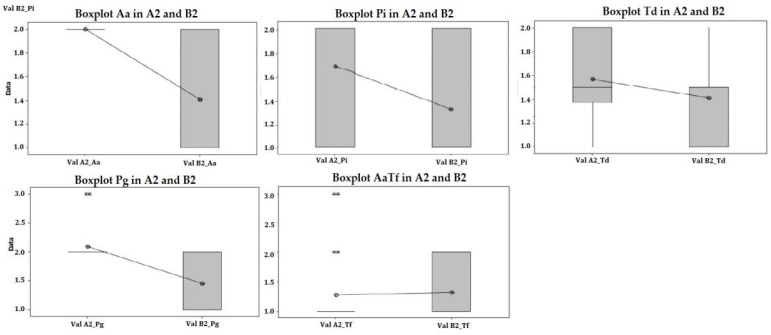
The Box plots show: *A. Actinomycetemcomitans*, *P. Gingivalis*-show statistically significant differences between groups; meanwhile, *P. Intermedia, T. Denticola and T. Forsythia* seem to show no significant difference between treatments.The median is the dot in the middle of the ‘box’. The upper side of the box is the 1st quartile or 25% of the data, from the rest. The lower side of the box is the 3rd quartile, or 75% of the data, from the rest. The distance between the sides of the box is called the inter-quartile range (IQR). The distance between these is called the range.

These results confirm the hypothesis that the use of aPDT as an adjunctive method to conventional treatment brings a benefit. Observing the raw data regarding the laboratory results, we consider it useful to highlight which of the five microorganisms included in the study are more susceptible to this method (Figure 10). Our results emphasize that aPDT has a better potential for bacterial elimination against *A. actinomycetemcomitans and P. gingivalis.* No significant bacterial elimination was observed between groups for the other three pathogens.

## 4. Discussion

Various complementary methods, such as systemic and local application of antibiotics, have been explored to complement the arsenal of mechanical debridement. However, many types of bacteria are resistant to antibiotics due to the biofilm structure of dental plaque. Systemic administration has several disadvantages, including a lack of drug concentration in the periodontal crevicular fluid (GCF), disruption of the intestinal flora, and the emergence of antibiotic-resistant bacteria [30]. For this reason, it is crucial to improve the effectiveness of periodontal treatment through the development of new antibacterial therapy modalities methods such as aPDT. 

This clinical study was conducted in order to evaluate the efficacy of aPDT as an adjunctive treatment method to periodontal therapy and to evaluate the microbiological effects of the use of photodynamic therapy in non-surgical periodontal treatment.

The idea behind the use of aPDT in the treatment of inflammatory dental problems is that a photosensitizer, typically a phenothiazine compound, can be taken up predominantly by bacteria and then activated by light of the appropriate wavelength in the presence of oxygen to start producing singlet-Oxygen and reactive species that are highly toxic to microorganisms [31]. These toxic species can cause cell death by damaging plasma membranes and DNA. Oxidative stress, inactivation of the membrane transport system, and inhibition of cell membrane enzyme activity are just a few ways in which cell membranes can be damaged. Without antibiotics or invasive procedures, antimicrobial PDT effectively reduces inflammation with minimal risk to the patient. Because the photosensitizer in aPDT is activated by cold laser light, it poses no threat to living tissue and it can even be used on dental restorations. 

On the other hand, the use of thermal lasers for subgingival curettage can cause surface damage to roots and carbonization of soft tissues, both of which can impede the reattachment of the surface epithelium [32]. Many studies investigating the use of diode lasers in the treatment of chronic periodontitis have been published, with better and better outcomes. It was difficult to compare the results of these studies due to variations in the laser wavelengths used and the clinical parameters assessed [33] and also the majority of these studies focused primarily on clinical outcomes, poorly assessing the changes in bacterial load in the periodontal pockets after immediate laser application [34].

According to the obtained results, the use of aPDT in a single application has been shown to be effective in treating periodontal disease in patients, thus succeeding in reducing the quantity of the majority of the periodontal pathogens. 

During this study, we observed a reduction in the total number of viable bacteria in periodontal pockets after the application of photodynamic therapy. To assess microbial reduction, we used the same tooth as a control, since the analysis included the total number of bacteria before/after scaling and before/after scaling and photodynamic therapy by comparison between the dependent groups. The clinical application of aPDT was tested as an adjunct to SRP for the treatment of chronic periodontitis. Conventional SRP does not completely eliminate periodontal pathogens residing in areas inaccessible to periodontal instruments [35]. These limitations could be attributed to several factors, such as the tooth anatomy, curettes‘ shape, and size or possible recolonization of periodontal pockets from other diseased sites or intraoral niches [36]. Antimicrobial photodynamic therapy has many advantages, such as a very low risk of developing photo resistance species or inducing mutagenic effects, even after multiple treatments. It also has broad-spectrum activity against Gram+ and Gram− bacteria. 

It has been reported that aPDT can kill microbial cells rapidly, especially when compared to antibiotics and antifungals, which can take days to take effect, while allowing selectivity for microorganisms over host tissues [37,38]. aPDT can be a useful therapeutic approach as the biofilm on the root surface can be easily reached with the dye and illuminated with light [39], then mechanical instruments hitting the “barrier” of the tooth root anatomy. 

The results published by Doertbudak et al., who took bacterial samples from peri-implantitis sites before and after aPDT treatment and cultured *A. actinomycetemcomitans*, *P. gingivalis* and *P. intermedia*, showed a reduced bacterial count by two logarithmic levels on average; however, complete elimination of bacteria was not possible [40]. A study published by De Oliveira et al. reported similar results to our study: the mean microbial concentrations decreased significantly in both study groups, but the results are difficult to compare because parameters and protocols are slightly different. In another in vitro study, the same research team evaluated the use of aPDT in oral bacteria and showed that the combination of a photosensitizer with low-power laser irradiation was effective in reducing *Aa*, *Pg,* and *Fn*. Therefore, it can be concluded that the use of other antimicrobial therapies such as photodynamic therapy and laser can be used as an adjunct to SRP [41]. Rhemrev et al. [42] investigated the reduction in bacterial counts directly after subgingival debridement by culture. Significant reductions were found for *T. forsythia*, *P. micra*, *F. nucleatum,* and spirochetes. No reductions were observed in *A. actinomycetemcomitans*, *P. gingivalis*, *P. intermedia,* and *C. rectus*.

Ruehling et al. did not find any additional significant improvement with photodynamic therapy in terms of PD, they reported that the microbial count was reduced by approximately 30% to 40% immediately after debridement and aPDT, returning to baseline at month 3, regardless of treatment [43]. Pinheiro et al. also showed a significantly greater reduction in the percentage of viable bacteria in periodontal pockets treated with aPDT (96%) compared to those treated with SRP alone (81%) [44].

Analysis of these cultures by polymerase chain reaction (PCR) using specific primers for particular strains of bacteria can allow the determination of which bacteria are more sensitive to photodynamic therapy and which are more resistant to this therapeutic approach. Yilmaz et al. reported short-term microbiological and clinical outcomes of treatment with soft laser in conjunction with methylene blue and/or SRP in ten patients. Within the limitations of this study, aPDT offered no additional microbiological and clinical advantages over traditional mechanical debridement [45]. Chondros et al. reported a statistically significant reduction of *Fusobacterium nucleatum* and *Eubacterium nodatum* in the test group at month 3. The levels of the microorganisms investigated also in our study (Aa, Pg, Tf, Td) were not significantly different [46].

Teodoro et al. evaluated the long-term clinical and microbiological effects of aPDT in the context of nonsurgical periodontal treatment in 33 patients. Although no statistically significant benefit in terms of clinical outcome could be demonstrated, aPDT treatment resulted in a significant reduction in the percentage of sites positive for all bacteria compared to SRP alone [47,48]. A systematic review and meta-analysis [49] of the included studies, focused on SRP + aPDT, showed conflicting results, although all studies reporting results from microbiological investigations analyzed changes in periodontopathogenic bacteria, only one [50] showed a significant reduction of *Porphyromonas gingivalis*. This finding is consistent with the results of an in vitro study that demonstrated the ability of aPDT to reduce levels of *Porphyromonas gingivalis*. Another study found significant differences in bacterial loads for *Fusobacterium nucleatum* and *Eubacterium nodatum* at 3 months and for *Eikenella corrodens* and *Capnocytophaga* species at 6 months, but reported an increase in bacterial loads for *Treponema denticola* in the aPDT-treated group [36].

As toluidine blue was used in conjunction with laser light, significant reductions in viable organism counts were achieved [51]. In 2019, two research groups reported significant results while using toluidine blue as antimicrobial therapy: Shen et al. investigated the antimicrobial effect of different toluidine blue concentrations, light irradiation, and duration on *Staphylococcus epidermidis* and *Staphylococcus aureus* isolated from ocular surface infection. They reported antibacterial efficacy when using a proper concentration of toluidine blue (60 μM) and certain laser parameters (5.27 mW/cm^2^ for 30 min) [52]. Anju and his research team were also interested in synthesizing a toluidine blue /multiwall carbon nanotube conjugate capable of cytotoxicity on *Pseudomonas aeruginosa* and *Staphylococcus aureus*. They obtained a photo inactivation of the bacteria included in the study, concluding that the conjugates may be used for the eradication of *P. aeruginosa* and *S. aureus* biofilms (58.49 J/cm^2^ for 3 min using a 670 nm red laser) [53]. In 2022, He et al. reported important results after investigating photo inactivation of methicillin-resistant *Staphylococcus aureus* using toluidine blue O. They studied different concentrations of toluidine blue and dosages of red-light laser radiation and concluded that biofilms showed shrinkage, fissure, fragmentation, and rarefaction after PDT treatment [54].

Ruhling et al. observed that aPDT with conventional ultrasonic debridement had a similar benefit in persistent periodontal pockets with a probing depth of at least 4 mm. They concluded that aPDT is not a superior treatment modality and should be used in conjunction with routine mechanical treatment [43]. 

Sigusch et al. (2010) conducted a clinical study to evaluate the efficacy of aPDT after routine SRP in patients with *F. nucleatum* at sites of chronic periodontitis. They assessed clinical parameters including plaque index, BOP, erythema, gingival recession, probing depth, and CAL at four-time points (baseline, 1, 4, and 12 weeks). They observed a significant reduction in redness, probe depth, and attachment level in the aPDT group [55].

Our study group performed a systematic review, to evaluate other results about the effectiveness of photodynamic therapy (aPDT) for periodontitis in adults as a primary mode of treatment or as an adjunct to non-surgical treatment. Randomized controlled trials, and systematic reviews, in the last 10 years were identified, all of them having PDT compared to conventional non-surgical treatment such as FMD and medication. Data on changes in clinical and microbiological parameters were extracted. Screening, data abstraction, and quality assessment were conducted. PDT as an independent treatment or as an adjunct to SRP did not demonstrate major statistically or clinically significant advantages, but combined therapy of PDT + SRP indicated a probable efficacy in CAL gain or probing depth reduction [56].

The results of the present study could have been more meaningful if a larger sample size and a longer follow-up time would have clarified the outcomes of this study. Lack of clinical parameters data due to no recall time, was another limitation; we intend to further assess changes in the clinical spectrum. Therefore, we consider that further clinical data acquisition and comparison will be valuable, as well as investigating other photosensitizers exposure to laser radiation.

Patients’ perspective of their oral health, treatment, and evolution seems to be significantly better when treated with a laser than in a conventional way. There is only a little evidence in the literature on how patients perceive other aspects of periodontal treatment procedures such as aPDT and it appears logical that patients’ perceptions should also be taken into account while evaluating treatment results, especially in chronic diseases such as periodontitis. Literature introduced long back the concept of the “patient-reported outcomes” umbrella term, which was proposed by the Food and Drug Administration (FDA) and represents “a measurement of any aspect of a patient’s health status that comes directly from the patient” [57]. In chronic illnesses that require maintenance, this could be a starting point for improving treatment outcomes, since clinicians can use the information regarding patient acceptance and assessment of different treatment methods [58,59]. 

On the other hand, repeated root planning may lead to some complications such as dental hypersensitivity and a certain loss of tooth structure, esthetic problems due to root exposure, and temporary slight mobility of the teeth [60], whereas the PDT ‘therapeutic window’ can be effective in reducing the biofilm/pathogens in areas difficult to access, with no such complications.

We, therefore, find interesting and very useful in the clinical practice the correlation between significant reduction in pathogens and the patients’ perception of a reduction in pain, together with the improvement of other clinical aspects. This could represent the basis for clinical protocols involving aPDT as a single treatment during periodontal maintenance, with improved patient compliance [61]. 

## 5. Conclusions

Our results suggest that aPDT can be considered a valuable adjunctive therapy, in initial periodontal treatment, enhancing the end result from a biological perspective. When comparing the decontamination potential of SRP alone with SRP + aPDT, it results in an increased decontamination potential of the combined treatment method, which was confirmed statistically. In our study, a single aPDT application proved an increased potential for bacterial elimination against *A. actinomycetemcomitans* and *P. gingivalis.* Although the PCR results testing at baseline and immediately after treatment, were not statistically different, greater germ count reductions were also observed for the other three pathogens: *P. Intermedia*, *T. Denticola,* and *T. Forsythia*. The authors agreed on the potential use of aPDT as a complementary treatment to conventional management of periodontitis. However, further studies are required inclining to the assessment of changes in PD, BoP, and clinical attachment level (CAL), as well as new perspectives on improving the interaction between different photosensitizers and laser radiation protocols.

Patients expressed greater satisfaction with combined treatment (SRP + aPDT), especially at 48 h postoperatively, when anticipatory stress no longer influences their responses. This was confirmed through significant statistical differences between treatment groups and it represents an encouraging factor regarding patient compliance in chronic diseases like periodontitis, which require maintenance.

## Data Availability

Not applicable.

## Abbreviations

PD	Pocket Depth- depth of the Periodontal Pockets in millimeters.
CAL	Clinical Attachment Level- measurements in millimeters.
BoP	Bleeding on Probing
SRP	Scaling and Root Planing
aPDT	antimicrobial Photodynamic Therapy
Nd:YAG	Neodymium-Doped Yttrium Aluminum Garnet
Aa	Aggregatibacter Actinomycetemcomitans
Pg	Porphyromonas Gingivalis
Pi	Prevotella Intermedia
Td	Treponema Denticola
Tf	Tannerella Forsythia
DNA	Deoxyribonucleic Acid
PCR	Polymerase Chain Reaction

## Appendix A

Questionnaire Form—Impact of treatment.
